# Exploring the evolutionary mechanism of hepatitis B and gastric cancer based on Mendelian randomization combined with bioinformatics analysis

**DOI:** 10.1097/MD.0000000000037645

**Published:** 2024-04-05

**Authors:** Huilian Cai, Tianjian Huang, Bohui Zheng, Xianqiong Zhu, Lisi Zhou, Jiayu Wu, Ying Xu, Shulan Huang, Yuxuan Huang, Tian Liu

**Affiliations:** a Guangzhou University of Chinese Medicine, Guangzhou, China; b General Hospital of Guangzhou Military Command of PLA, Guangzhou, China; c Guangdong Pharmaceutical University, Guangzhou, China; d Foshan Hospital of Traditional Chinese Medicine, Foshan, China.

**Keywords:** causal inference, extrahepatic cancers, hepatitis B, Mendelian randomization

## Abstract

Chronic hepatitis B virus infection (HBV) infection appears to be associated with extrahepatic cancers. This study aims to evaluate the causality and evolutionary mechanism of chronic HBV infection and gastric cancer through Mendelian randomization (MR) analysis and bioinformatics analysis. We conducted 2-sample MR to investigate the causal relationship between chronic HBV infection and gastric cancer. We identified 5 independent genetic variants closely associated with exposure (chronic HBV infection) as instrumental variables in a sample of 1371 cases and 2938 controls of East Asian descent in Korea. The genome wide association study (GWAS) data for the outcome variable came from the Japanese Biobank. Bioinformatics analysis was used to explore the evolutionary mechanism of chronic HBV infection and gastric cancer. Differential expression analysis and weighted gene co-expression network analysis (WGCNA) were performed to identify key targets that are commonly associated with both diseases, and their biological functions were investigated. Multiple machine-learning models were employed to select hub genes. The MR analysis showed a positive causal relationship between chronic HBV infection and gastric cancer (IVW: OR = 1.165, 95% CI = 1.085–1.250, *P* < .001), and the result was robust in sensitivity analysis. According to the bioinformatics analysis, the 5 key targets were mainly enriched in Toll-like receptor signaling and PI3K-Akt signaling. Two hub genes, CXCL9 and COL6A2, were identified, and a high-performing predictive model was constructed. Chronic HBV infection is positively associated with gastric cancer, and the evolutionary mechanism may be related to Toll-like receptor signaling. Prospective studies are still needed to confirm these findings.

## 1. Introduction

As research on the “inflammation-cancer” transformation deepens, the impact of hepatitis B virus infection (HBV) on cancer has attracted increasing attention. It is estimated that approximately 360 million people worldwide are infected with HBV.^[1,2]^ Previous studies have indicated that chronic HBV infection is a risk factor for hepatocellular carcinoma^[3]^ and is associated with extrahepatic cancers such as pancreatic, lung, gastric, and thyroid.^[4,5]^ However, the results above are based on observational studies with limited evidence, and the causal relationship between chronic HBV infection and extrahepatic cancer remains uncertain. Moreover, these studies may be influenced by confounding factors. Therefore, further research using alternative methods is needed to explore these associations.

The Mendelian randomization (MR) analysis improves causal inference by utilizing genetic variation as an instrumental variable to infer the causal relationship between exposure and outcomes.^[6]^ Unlike observational studies, MR analysis is not subject to confounding by unmeasured factors due to the random independent assortment of alleles during meiosis.^[7,8]^ Furthermore, as genetic variants are fixed at birth and do not change over time, MR analysis is less susceptible to reverse causation.^[9]^ MR is an ideal method for inferring causal relationships between exposure and outcomes.

Bioinformatics has become a critical tool in screening disease biomarkers in modern medical research. This is primarily due to its ability to effectively handle large amounts of data generated by high-throughput experimental techniques and integrate biological data from different sources, thereby providing more comprehensive information. Bioinformatics offers a systematic perspective for analyzing the relationship between biomarkers and diseases, making it faster and more cost effective than traditional laboratory methods. This approach guides the subsequent validation of experiments and identifies biomarkers specific to individuals or subsets of populations, thus contributing to the development of personalized medicine.

There has been no research exploring the causal relationship between HBV infection and gastric cancer, as well as their underlying mechanisms. Therefore, in this study, we hypothesize that there is a causal relationship between hepatitis and gastric cancer, and we validate this hypothesis using a bidirectional MR analysis. We then investigate the potential mechanisms underlying the association between hepatitis and gastric cancer by conducting bioinformatics analysis to identify biomarkers closely related to both diseases and explore their possible evolutionary mechanisms.

## 2. Methods and materials

### 2.1. MR analysis

#### 2.1.1. Data acquisition.

Genetic variations closely associated with chronic HBV infection were identified from a Korean East Asian population-based genome wide association study (GWAS) study comprising 1371 chronic HBV patients and 2938 control individuals. The study employed a selection process based on the lowest *P* value single nucleotide polymorphism (SNP), leading to the identification of instrumental variables significantly associated with chronic HBV infection (*P* < 5 × 10^−8^). Additionally, linkage disequilibrium was corrected for (r² < 0.001 and distance > 10000kb) to ensure the independence of the instrumental variables. In this study, palindrome SNPs were excluded. To avoid violating the third assumption of MR (horizontal pleiotropy), we used PhenoScanner to identify and exclude any genetic variations directly related to cancer at specific loci.^[10]^

Genetic variations associated with gastric cancer were obtained from a recent large-scale GWAS study based on the Japanese East Asian population (https://biobankjp.org/en/index.html). The study included 6563 patients and 195,745 control individuals. The detailed data included in MR analysis can be found in Table 1.

**Table 1:** Dataset in MR analysis.

Dataset	Application
Korean East Asian population-based GWAS study comprising 1371 chronic HBV patients and 2938 control individuals	MR analysis
GWAS study based on the Japanese East Asian population included 6563 patients and 195,745 control individuals	

GWAS = genome wide association study, HBV = hepatitis B virus infection, MR = Mendelian randomization.

#### 2.1.2. Statistical analysis.

We performed the MR analysis using the inverse variance weighted (IVW) method, which provides accurate estimates when there is no heterogeneity and directional pleiotropy between the exposure and outcome variables.^[11]^ Heterogeneity in the causal relationship between chronic HBV infection and gastric cancer was investigated by estimating Cochran Q statistic using a fixed-effects model.^[12]^ Sensitivity analysis was conducted to examine and correct for pleiotropy in the causal estimates.^[11]^ If the Q statistic was significant at *P* < .05, indicating the presence of heterogeneity, we employed the random-effects IVW method in our analysis.^[13]^ We assessed the presence of horizontal pleiotropy using MR-Egger regression.^[14]^ The presence of horizontal pleiotropy was indicated if the intercept of the MR-Egger regression deviated from zero or if its *P* value was statistically significant at *P* < .05.^[15]^ We also used the weighted median method as a complement in this analysis. The weighted median method can still provide valid estimates in horizontal pleiotropy. MR-PRESSO detected and removed outliers in instrumental variables.^[14]^ The above analysis process was conducted using the MR-PRESSO^[16]^ and TwoSampleMR^[17]^ packages.

### 2.2. Bioinformatics analysis

#### 2.2.1. Gene expression profile data.

The dataset used in this study was obtained from the GEO database (https://www.ncbi.nlm.nih.gov/geo/query/acc.cgi). The GSE83148 dataset, based on the GPL570 platform, consists of 122 samples with chronic HBV infection and 6 samples of normal liver tissue. Samples concurrently infected with HCV, fatty liver or chronic liver diseases, and chronic alcoholic hepatitis were excluded from this study. The GSE29272 dataset, based on the GPL96 platform, includes 134 gastric cancer samples and 134 normal control samples. The GEOquery^[18]^ package retrieved and standardized the data above. The detailed data included in bioinformatics analysis can be found in Table 2.

**Table 2:** Dataset in bioinformatics analysis.

Dataset	Application
The GSE83148 dataset, based on the GPL570 platform, consists of 122 samples with chronic HBV infection and 6 samples of normal liver tissue	Bioinformatics analysis
The GSE29272 dataset, based on the GPL96 platform, includes 134 gastric cancer samples and 134 normal control samples	

HBV = hepatitis B virus infection.

#### 2.2.2. Differentially expressed gene screening.

The matrix data were normalized using R software version 4.1.3. Differentially expressed genes (DEGs) were identified using the limma^[19]^ package, with the |logFC| ≥ 1 criterion and *P* < .05.

#### 2.2.3. Weighted gene co-expression network analysis (WGCNA).

WGCNA^[20]^ was used to classify genes into different modules based on their association and evaluate the relationship between modules and clinical features, such as disease, age, and gender, to determine the most relevant modules. This study employed WGCNA to identify modules most strongly associated with chronic hepatitis B infection and gastric cancer as separate observation variables. Genes within these modules were extracted and overlapped with DEGs to identify key genes commonly associated with chronic hepatitis B infection and gastric cancer.

#### 2.2.4. Functional enrichment analysis.

The gene ontology analysis is a standard method for large-scale functional enrichment studies, covering biological processes, molecular functions, and cellular components. The Kyoto Encyclopedia of Genes and Genomes (KEGG) is a widely used database for storing information on genomics, biological pathways, diseases, and drugs.^[21]^ This study performed differential gene expression and enrichment analysis of gene ontology annotations and KEGG pathways using the clusterProfiler^[22]^ package in R. A threshold of FDR < 0.05 was considered statistically significant.

#### 2.2.5. Construction and validation of prediction models.

The GSE83148 and GSE29272 datasets were divided into training sets (70%) and test sets (30%). Four common classifiers, namely Support Vector Machine (SVM), Random Forest (RF), Extreme Gradient Boosting, and Generalized Linear Model, were constructed using the training set, and 10-fold cross-validation was performed based on the “care” package.^[23]^ Subsequently, each model Area Under the Curve (AUC) value was calculated, and the model with the highest AUC value was determined to have the best performance. The top 3 genes were extracted based on the importance of these models, and the intersection of these genes was identified as hub genes. Using the best-performing model, a prediction model was reconstructed using only the hub genes in the training set, and this model performance was validated using the test set. The RMDA^[24]^ package was utilized to generate DCA curves for the models and assess the clinical value of the hub genes.

## 3. Results

### 3.1. MR results

**Figure 1.**
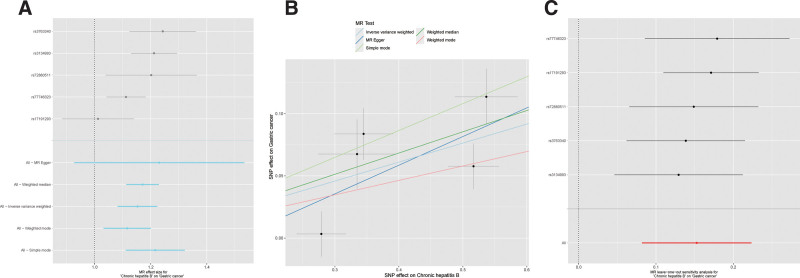
MR analysis. (A) MR analysis forest plot. (B) MR analysis scatter plot. (C) Leave-one-out analysis. MR = Mendelian randomization.

This study extracted 5 SNP variants closely associated with chronic hepatitis B virus (HBV) infection. Additionally, after PhenoScanner identification, all instrumental variables were unrelated to gastric cancer. The general information on the instrumental variables is presented in Table 3. The IVW method, our primary MR approach without horizontal pleiotropy, revealed a causal relationship between chronic HBV infection and gastric cancer (OR = 1.165, 95% CI = 1.085–1.250, *P* < .001, Fig. 1A). The simple model (OR = 1.241, *P* = .019), weighted model (OR = 1.123, *P* = .048), weighted median (OR = 1.186, *P* < .001), and MR-Egger (OR = 1.259, *P* = .234) all showed consistent effects with the IVW approach (OR > 1, Fig. 1B). Therefore, the MR analysis results provide evidence of a positive causal relationship between chronic HBV infection and gastric cancer. Sensitivity analysis results indicated heterogeneity, as evidenced by Cochran Q test (IVW Q test: *P* = .032). Hence, the random-effects IVW method was adopted as the primary MR approach. The MR-Egger regression intercept was close to zero (b = −0.033, *P* = .640). MR-PRESS analysis demonstrated that none of the included SNP variants exhibited outliers in this study. Leave-one-out analysis demonstrated consistent MR results with the inclusion of all SNPs, indicating minimal changes in the results (Fig. 1C).

**Table 3:** General information on the instrumental variables.

SNP	EA	EAF	Related to chronic hepatitis B			Related to gastric cancer		
			b	se	*P*	b	se	*P*
rs17191293	G	0.459	0.279	0.039	5.90745E-13	0.003	0.018	.857
rs3134993	A	0.705	0.537	0.049	6.03254E-28	0.114	0.022	.000
rs3763340	C	0.240	−0.345	0.046	4.83281E-14	−0.084	0.021	.000
rs72880511	A	0.115	0.335	0.061	3.83557E-08	0.067	0.028	.015
rs77746323	T	0.599	0.517	0.040	1.32221E-38	0.058	0.018	.002

SNP = single nucleotide polymorphism.

### 3.2. Differential expression analysis

**Figure 2.**
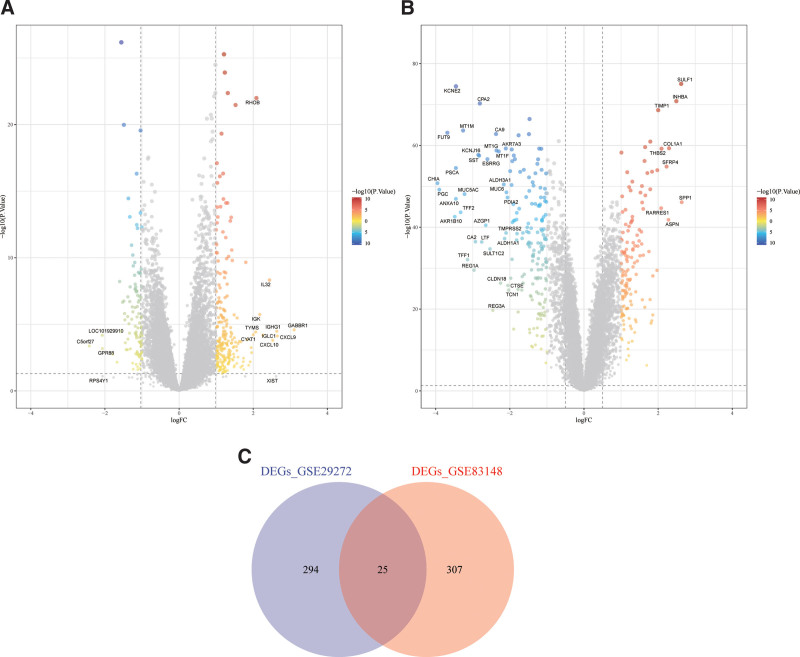
Differential expression analysis. (A) GSE83148 differential expression analysis. (B) GSE29272 differential expression analysis. (C) Venn diagram.

In the GSE83148 dataset, we identified 332 DEGs, consisting of 227 upregulated and 105 downregulated genes (Fig. 2A). In the GSE29272 dataset, we identified 319 DEGs, including 137 upregulated and 182 downregulated genes (Fig. 2B). 25 genes overlapped between the 2 datasets (Fig. 2C).

### 3.3. WGCNA

**Figure 3.**
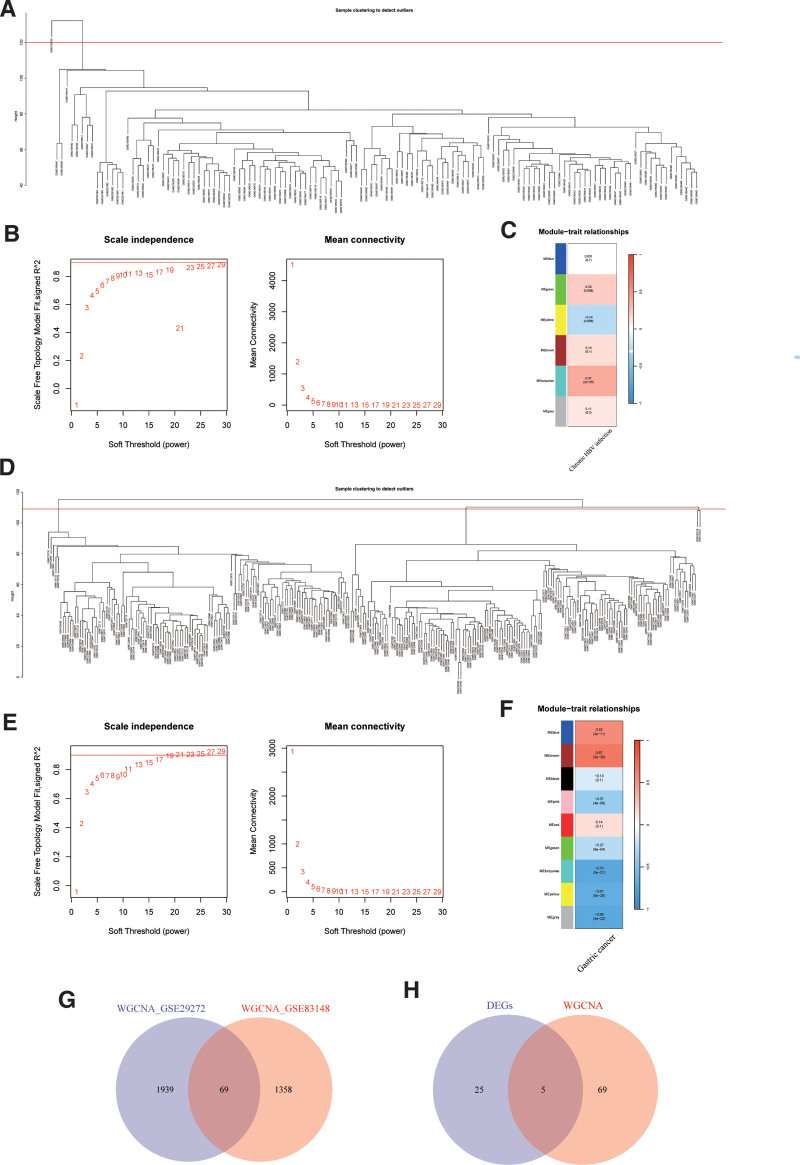
WGCNA results. (A) GSE83148 sample clustering plot. (B) Soft thresholding selection. (C) Heatmap of associations with chronic HBC infection traits. (D) GSE29272 sample clustering plot. (E) Soft thresholding selection. (F) Heatmap of associations with gastric cancer traits. (G) Venn diagram of genes in the most correlated WGCNA module. (H) Venn diagram of overlapping DEGs and WGCNA results. DEGs = differentially expressed genes, WGCNA = weighted gene co-expression network analysis.

Based on GSE83148, we removed the outlier sample GSM2195535 (Fig. 3A). We successfully constructed a scale-free network when the soft threshold β was set to 23 (Fig. 3B). The turquoise module was closely associated with the disease state (correlation = 0.37, *P* = 2e-5, Fig. 3C). This module included 1427 genes. Based on GSE29272, we removed the outlier samples GSM723519 and GSM723521 (Fig. 3D). A scale-free network was constructed with a soft threshold β of 17 (Fig. 3E). The turquoise module was closely associated with the disease state (correlation = −0.70, *P* = 2e-21, Fig. 3F). This module included 2008 genes, with 69 overlapping genes between the 2 modules (Fig. 3G). Finally, we identified 5 key genes (Fig. 3H) through differential expression analysis and WGCNA: COL6A2, PDGFD, SPP1, CXCL9, and CXCL8.

### 3.4. Functional enrichment analysis

**Figure 4.**
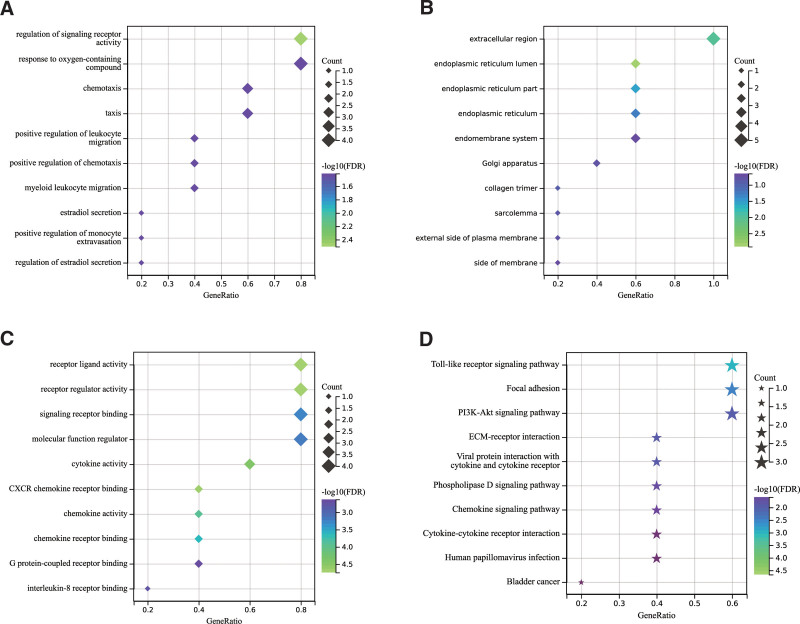
Enrichment analysis. (A) Biological process (BP). (B) Cellular component (CC). (C) Molecular function (MF). (D) KEGG pathways. KEGG = Kyoto encyclopedia of genes and genome.

Functional enrichment analysis was conducted, and the results were filtered based on a threshold of FDR < 0.05. In the biological process category, key genes were mainly enriched in processes such as regulation of signaling receptor activity, response to oxygen-containing compound, and chemotaxis. In the cellular component category, key genes were primarily enriched in the extracellular region, endoplasmic reticulum lumen, and endoplasmic reticulum part. In the molecular function category, key genes showed enrichment in receptor ligand activity, receptor regulator activity, and signaling receptor binding. Regarding KEGG pathways, key genes were significantly enriched in Toll-like receptor signaling, PI3K-Akt signaling, and Phospholipase D signaling (Fig. 4).

### 3.5. Machine learning

**Figure 5.**
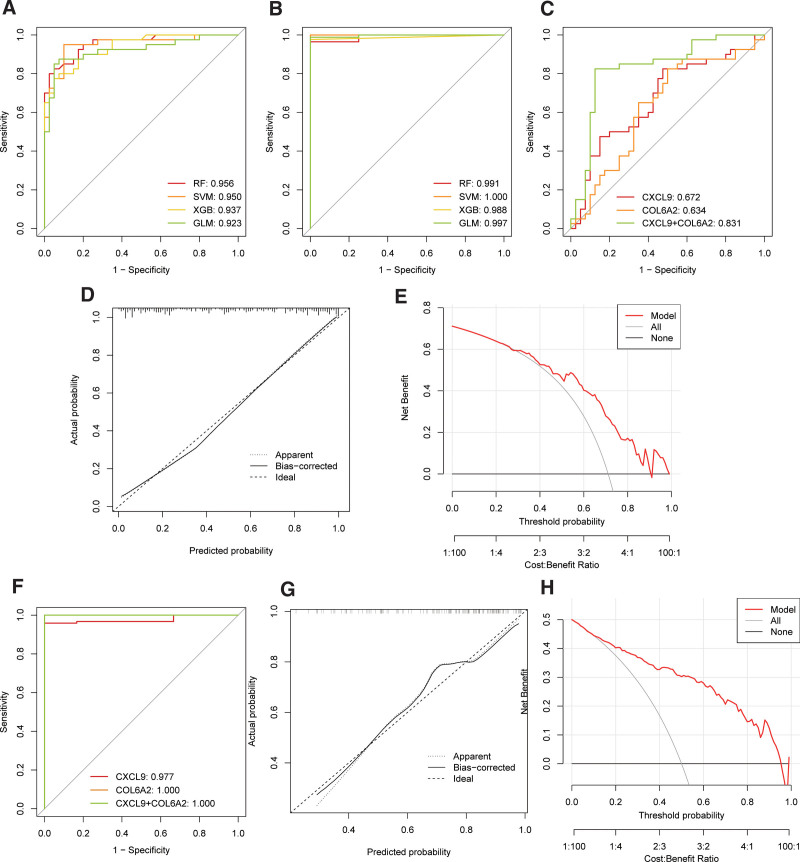
Machine learning. (A) Performance of different classifiers in GSE29272. (B) Performance of different classifiers in GSE83148. (C) Performance of hub genes in GSE29272. (D) Performance of hub genes in GSE83148. (E) Calibration curve of hub genes in GSE29272. (F) Decision curve analysis (DCA) curve of hub genes in GSE29272. (G) Calibration curve of hub genes in GSE83148. (H) Decision curve analysis (DCA) curve of hub genes in GSE83148.

In the GSE29272 training set, we constructed 4 classifier models based on key genes, with the RF model showing the best predictive performance (AUC = 0.956, Fig. 5A). Therefore, we considered the RF model optimal for the GSE29272 dataset and identified the top 3 target genes in the RF model importance ranking: CXCL8, CXCL9, and COL6A2. Similarly, in the GSE83148 training set, we built 4 classifier models based on key genes, with the SVM model demonstrating the best predictive performance (AUC = 1.000, Fig. 5B). Hence, we regarded the SVM model as the optimal model for the GSE83148 dataset and identified the top 3 target genes in the SVM model importance ranking: PDGFD, CXCL9, and COL6A2. The intersection analysis of the predicted target genes from both datasets yielded 2 common target genes: CXCL9 and COL6A2, considered hub genes.

We reconstructed the RF prediction model on the GSE29272 training set using the hub genes. The results showed excellent predictive performance on the test set (AUC: 0.831, Fig. 5C), and the calibration curve confirmed the model good performance (Fig. 5D). Additionally, through the clinical decision curve, the model demonstrated high clinical net benefit (Fig. 5E).

Similarly, we constructed the SVM prediction model on the GSE83148 training set using the hub genes. The results also exhibited excellent predictive performance on the test set (AUC: 1.000, Fig. 5F), and the calibration curve confirmed the model good performance (Fig. 5G). Through the clinical decision curve, the model demonstrated high clinical net benefit (Fig. 5H).

## 4. Conclusion

Although HBV is considered a hepatotropic virus, some observational evidence suggests an association between chronic HBV infection and extrahepatic cancers, including esophagal, endometrial, cervical, gastric, lung, and colorectal cancer. Given the limitations of observational epidemiological studies in establishing causality, we propose a scientific hypothesis that chronic HBV infection may contribute to gastric cancer development through certain mechanisms. To demonstrate this hypothesis, we will employ MR analysis to determine the causal relationship between the 2 and conduct a combined bioinformatics analysis to explore the underlying mechanisms. This is the first comprehensive investigation using MR and bioinformatics analysis to explore the potential and mechanisms of gastric cancer development associated with chronic HBV.

Through MR analysis, we have established a causal relationship between chronic HBV infection and gastric cancer. It is widely believed that HBV protein functions as a transcriptional coactivator, playing a crucial role in tumor initiation by modulating key regulators of cellular apoptosis and interfering with DNA repair pathways and tumor suppressor genes. Additionally, Song et al^[25]^ observed high expression of HBV protein in the cancerous sections of tissue specimens compared to the healthy sections of the same specimens, providing further support for the carcinogenic effect of HBV protein. Furthermore, some studies have found the presence of HBV DNA in liver-extrinsic tissues, including the stomach, kidneys, gallbladder, and pancreas, indicating that HBV can initiate and promote tumor development outside the liver.^[26,27]^

During the bioinformatics analysis, we combined differential expression analysis and WGCNA to identify 5 key targets closely associated with chronic HBV infection and gastric cancer, which are considered critical targets for progressing chronic HBV infection to gastric cancer. Through enrichment analysis, these targets were mainly enriched in pathways such as Toll-like receptor signaling, PI3K-Akt signaling, and Phospholipase D signaling. Subsequently, we further employed multiple machine learning algorithms to screen out 2 hub genes and constructed prediction models based on these hub genes, which demonstrated the best performance in prediction accuracy, generalization, calibration, and net clinical benefit. These models are of great clinical significance and deserve attention.

Toll-like receptors (TLRs) are a crucial group of innate immune receptors that recognize corresponding molecular patterns of pathogens, activating downstream adapter molecules MyD88 and TRIF, which in turn activate NF-κB, MAPK, and IRF signaling pathways, inducing the production of inflammatory factors and type I interferons, thus activating innate and acquired immune responses.^[28]^ HBV infection downregulates the expression and function of host TLRs, evading immune surveillance, for instance, the expression of TLR2 and TLR3 is downregulated in peripheral blood mononuclear cells of patients with chronic HBV infection.^[29]^ TLR gene polymorphisms are associated with HBV infection outcomes, such as TLR2 gene polymorphisms being associated with HBV viral load and disease activity.^[30]^ Activation of TLR enhances the anti-HBV immune response, as TLR3 agonist poly(I:C) promotes HBV-specific T cell response, clearing HBV.^[31]^ TLR2 priming reduces the number of liver macrophages, increases the number of dendritic cells, and enhances HBV-specific T cell response.^[32]^ In summary, the TLR signaling pathway plays a crucial role in the HBV infection immune response, and activation of TLR signaling enhances the anti-HBV immune response. Furthermore, the correlation between Toll-like receptor signaling pathway and gastric cancer has also been confirmed, with increased expression of TLR4, TLR5, and TLR9 in gastric cancer tissues being associated with the occurrence of gastric cancer.^[33]^ Activation of TLR signaling promotes the production of inflammatory factors like IL-6 and TNF-α, which are involved in the development of hepatitis B and gastric cancer.^[34]^ Imbalance in TLR signaling may lead to chronic HBV infection and the occurrence of gastric cancer, and moderate activation of TLR may have therapeutic effects, but the specific mechanism still needs further investigation.

The association between chronic hepatitis B infection and extrahepatic cancers is gradually receiving clinical attention. As the first comprehensive analysis combining MR and bioinformatics, this study investigates the causal transformation mechanism between chronic hepatitis B infection and gastric cancer. It confirms the risk of gastric cancer induction by chronic hepatitis B infection, which may be associated with the TLR signaling pathway mediated by CXCL9 and COL6A2. Additionally, a high-performance prediction model was constructed, highlighting its clinical significance. However, our study has certain limitations. Firstly, the GWAS studies were all derived from East Asian populations, and whether a causal relationship exists in European populations remains unknown. Secondly, the MR assumption assumes a linear relationship between exposure and outcome. Thus, if the relationship between depression and cancer is non-linear, it may not be applicable.^[35]^ Therefore, further prospective studies are still needed to validate our findings.

## Author contributions

**Conceptualization:** Huilian Cai.

**Data curation:** Huilian Cai.

**Formal analysis:** Huilian Cai, Lisi Zhou.

**Investigation:** Xianqiong Zhu.

**Methodology:** Tianjian Huang, Bohui Zheng, Xianqiong Zhu, Lisi Zhou, Ying Xu.

**Supervision:** Tian Liu.

**Visualization:** Xianqiong Zhu, Jiayu Wu, Ying Xu, Yuxuan Huang.

**Writing – original draft:** Huilian Cai, Tianjian Huang, Bohui Zheng, Xianqiong Zhu, Jiayu Wu, Ying Xu, Yuxuan Huang, Tian Liu.

**Writing – review & editing:** Lisi Zhou, Jiayu Wu, Shulan Huang, Tian Liu.
